# NOGO-A/RTN4A and NOGO-B/RTN4B are simultaneously expressed in epithelial, fibroblast and neuronal cells and maintain ER morphology

**DOI:** 10.1038/srep35969

**Published:** 2016-10-27

**Authors:** Olli Rämö, Darshan Kumar, Erika Gucciardo, Merja Joensuu, Maiju Saarekas, Helena Vihinen, Ilya Belevich, Olli-Pekka Smolander, Kui Qian, Petri Auvinen, Eija Jokitalo

**Affiliations:** 1 Cell and Molecular Biology Program, Institute of Biotechnology, University of Helsinki, Helsinki, Finland.; 2 Electron Microscopy Unit, Institute of Biotechnology, University of Helsinki, Helsinki, Finland.; 3 DNA Sequencing and Genomics Laboratory, Institute of Biotechnology, University of Helsinki, Helsinki, Finland

## Abstract

Reticulons (RTNs) are a large family of membrane associated proteins with various functions. NOGO-A/RTN4A has a well-known function in limiting neurite outgrowth and restricting the plasticity of the mammalian central nervous system. On the other hand, Reticulon 4 proteins were shown to be involved in forming and maintaining endoplasmic reticulum (ER) tubules. Using comparative transcriptome analysis and qPCR, we show here that *NOGO-B/RTN4B* and *NOGO-A/RTN4A* are simultaneously expressed in cultured epithelial, fibroblast and neuronal cells. Electron tomography combined with immunolabelling reveal that both isoforms localize preferably to curved membranes on ER tubules and sheet edges. Morphological analysis of cells with manipulated levels of NOGO-B/RTN4B revealed that it is required for maintenance of normal ER shape; over-expression changes the sheet/tubule balance strongly towards tubules and causes the deformation of the cell shape while depletion of the protein induces formation of large peripheral ER sheets.

Two members of reticulon (RTN) family, NOGO-A/RTN4A and NOGO-B/RTN4B, have recently been the focus of intense investigation due to their functions as an inhibitor of neurite outgrowth and involvement in restricting the plasticity of the central nervous system^1^,^2^,^3^ and on the other hand, in generating curvature on ER tubules^4^. The discrepancy between these findings comes from the required localization and topology of membrane insertion needed to support these functions. It is difficult to explain how one protein, or structurally very similar isoforms, can be localized on the cytosolic side of the ER membrane and on extracellular side of the plasma membrane (PM)^5^.

Mammals have four reticulon genes (*RTN1*, *RTN2*, *RTN3* and *NOGO/RTN4*), each of which, due to differential splicing and promoter usage, can give rise to a range of alternatively spliced transcript variants that are encoding different isoforms^5^. Most transcripts are enriched in nervous tissues. *NOGO-A/RTN4A* has been regarded as a neuron specific form, whereas NOGO-B/RTN4B has a widespread expression pattern, as in case of housekeeping genes^5^. The *RTN* family is characteristic for its highly conserved C-terminal reticulon homology domain (RHD) of 150–200 amino acids containing two hydrophobic stretches separated by a 66 amino-acid hydrophilic loop and followed by a short C-terminal tail^6^. In comparison to the closely conserved C-terminus that may give rise to overlapping functions within the RTN family, no sequence homology can be observed in the N-terminus of the variants^5^.

Rapoport and colleagues demonstrated that *NOGO-A/RTN4A* together with DP1 (deleted in polyposis 1, also known as receptor expression enhancing protein 5, REEP5^7^; yeast homolog *Yop1p*^8^) is responsible for generating and maintaining ER tubules^4^. Overexpression of these proteins generates long unbranched tubules, whereas their deletion leads to loss of tubular ER in yeast *Saccharomyces cerevisiae*^4^. Authors suggested that the hydrophobic regions of reticulon homology domain may form two hairpin loops that are wedged into the outer membrane leaflet, creating the necessary increase in local surface area needed for high membrane curvature. Local, one-dimensional curvature is extended and stabilized by the formation of extensive homo- and hetero-oligomers between reticulons and DP1/Yop1p generating a scaffold within the membrane along the tubule’s length^9^.

The formation of reticular ER network of tubules requires interplay between several other proteins. REEPs, a family of membrane associated proteins, have been shown to affect ER structure^4^,^7^,^9^,^10^ along with connecting ER tubules to cytoskeleton. Like other ER related proteins, the mutations in REEP1 were found to be associated with neurodegenerative disorder, hereditary spastic paraplegia (HSP)^11^,^12^. The atlastin (1–3) proteins (and their yeast homolog Sey1p) belong to a large protein family that interact with the reticulons and DP1/Yop1p and stimulate homotypic fusion, *e*.*g*., membrane fusion between ER tubules or sheets, to produce branched reticular ER network^9^,^13^,^14^. Mutations in or depletion of atlastins in mammalian cells leads to long unbranched ER tubules, and to network fragmentation in fruit fly *Drosophila melanogaster* neurons, while the over-expression leads to ER membrane expansion^14^,^15^. Atlastin has been shown to bind to ATPase spastin^16^ that interacts with RTN1^17^.

In the present study, we have performed a comparative transcriptome analysis and quantitative PCR (qPCR) for expression profiling of the whole reticulon family in cultured human hepatoma and mouse fibroblast cell lines and primary mouse neurons, and show that *NOGO-B/RTN4B* is the main *RTN4* isoform expressed in hepatoma and fibroblast cells and *Nogo-A/Rtn4A* in primary neurons. However, in all cell types studied, several of the other *RTN4* isoforms are expressed at reasonable high levels too, suggesting that none of the isoforms should be regarded as a cell type specific isoform. High resolution imaging and localization studies revealed that both NOGO-A/RTN4A and NOGO-B/RTN4B localized on ER. We have been unable to find evidence for RTN4 plasma membrane localization. Using electron tomography (ET) combined with immunolabelling, we were able to show that both proteins localized preferably to curved membranes on ER tubules and sheet edges. Morphological analysis of cells with manipulated levels of NOGO-A/RTN4A or NOGO-B/RTN4B revealed that these proteins are required for maintenance of normal ER shape; over-expression changes the sheet/tubule balance strongly towards tubules and causes the deformation of the cell shape while depletion induces formation of large peripheral ER sheets.

## Results

### Several reticulon 4 splice variants are simultaneously expressed in cultured human Huh-7, mouse NIH/3T3 and mouse primary neuronal cells

**Figure 1:**
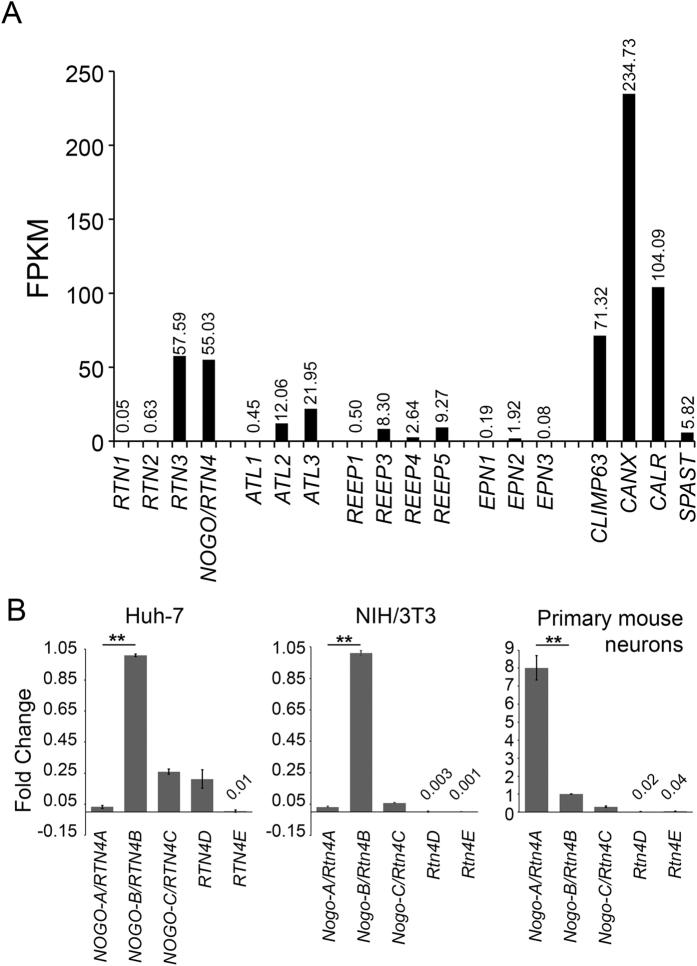
Several reticulon 4 splice variants are simultaneously expressed in cultured human hepatoma (Huh-7), mouse fibroblast (NIH/3T3) and primary mouse neuronal cells. (**A**) Transcriptome showing fragments per kilobase of transcript per million mapped reads (FPKM) values for indicated mRNA levels in Huh-7 cells. (**B**) qPCR data showing relative mRNA levels for indicated *RTN4* isoforms in Huh-7, NIH/3T3 and primary mouse cortical neurons. Species specific β-actin was used as internal controls. Graphs in B were normalized against *NOGO-B/RTN4-B* for all three cell types.

As a starting point for the present study, we performed a comparative transcriptome analysis to study the expression of all *RTN* family members in human hepatoma cell line (Huh-7). For this, we extracted total mRNA for SOLiD sequencing^18^,^19^ from where the reads were mapped to compare the expression levels of *RTN* family members and other ER-related proteins. The analysis revealed that although all four *RTN* genes were simultaneously expressed, expression levels of *RTN3* and *RTN4* were equal and clearly exceeded those of *RTN1* and *RTN2*. Compared to the genes encoding ER structure related proteins, *RTN3* and *RTN4* levels were only slightly lower than ER sheet promoting *CLIMP63*, and clearly higher than *ATL* (encoding for atlastins) and *REEP*s. On the other hand, compared to the genes encoding ER chaperones *CANX* (encoding for calnexin) and *CALR* (encoding for calreticulin), the levels were about 4- and 2-fold lower, respectively (Fig. 1A).

*RTN4* gene gives rise to five isoforms, *RTN4A-E*. We used qPCR to determine the expression levels of the splice variants in Huh-7 cells, cultured mouse embryonic fibroblast (NIH/3T3) cells and in mouse primary neurons. First, all *RTN4* isoforms were expressed in Huh-7 cells; *NOGO-B/RTN4B* was the main isoform expressed, and its level was around 4-fold higher compared to *NOGO-C/RTN4C*, 5-fold to *RTN4D* and 30-fold to *NOGO-A/RTN4A*. In contrast, the expression of *RTN4E* was just at detectable level (Fig. 1B). In NIH/3T3 cells, *Nogo-B/Rtn4B* was also the main isoform expressed, and the ratio between A and B isoforms was similar to Huh-7 cells, whereas *Nogo-C/Rtn4C* levels were 17 times lower in NIH/3T3 cells. In mouse neurons, the expression profile was different; *Nogo-A/Rtn4A* was the main isoform and its level was about 8- and 27-folds higher compared to *Nogo-B/Rtn4B* and *Nogo-C/Rtn4C*, respectively (Fig. 1B). The expression of *Rtn4E* and *Rtn4D* were both very low (Fig. 1B). In summary, qPCR revealed that all reticulon 4 isoforms were expressed in human hepatoma cells as well as in mouse fibroblasts and primary neurons, but at varying levels. Comparison of the three cell types revealed clear difference between neurons and other cell types, whereas similar profile was found from non-neuronal cells of human and mouse origin. *NOGO-B/RTN4B* and *Nogo-A/Rtn4A* are the main isoforms in non-neuronal and neuronal cells, respectively. Since *NOGO-A/RTN4A* has reasonably high expression in non-neuronal cells too, it should not be considered purely as a neuron specific isoform.

### Endogenous reticulon 4B and 4A localize on ER membranes and show preference to positively curved membranes

**Figure 2:**
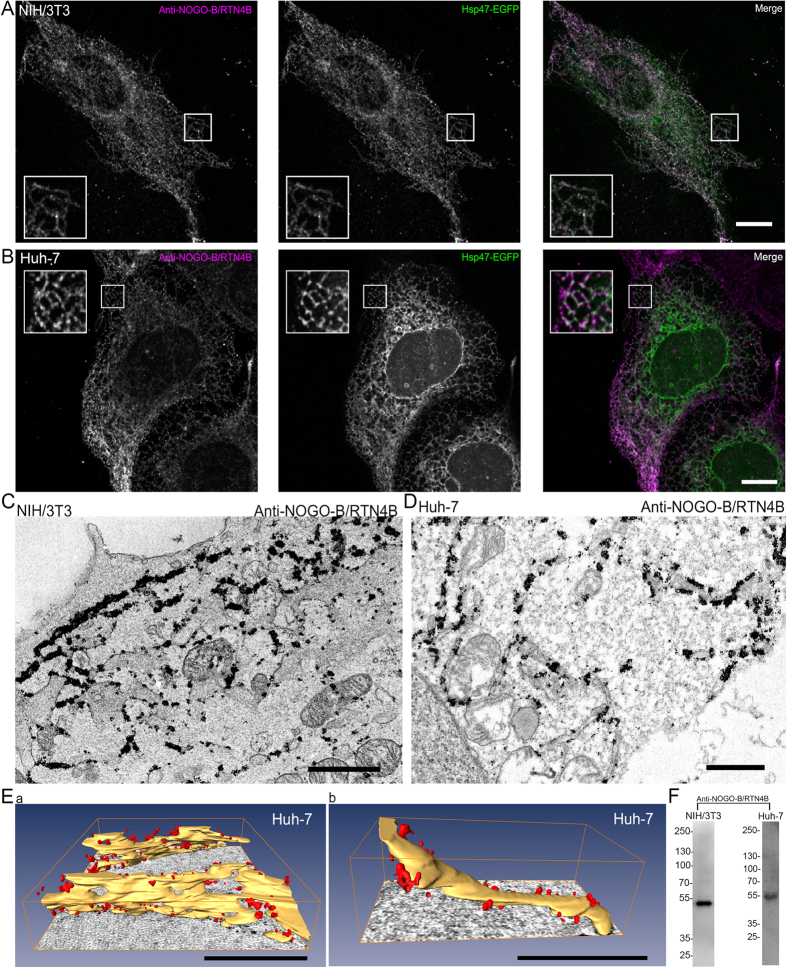
NOGO-B/RTN4B localizes to ER tubules and sheet edges. Confocal LM images of (**A**) NIH/3T3 and (**B**) Huh-7 cells showing localization of immunolabelled endogenous NOGO-B/RTN4B and ER marker Hsp47-EGFP in the ER. Merged images reveal co-localization in ER tubules and sheet edges. Insets show higher magnification of boxed areas. TEM micrographs of (**C**) NIH/3T3 and (**D**) Huh-7 cells showing immunolabelling of endogenous NOGO-B/RTN4B. (**E**) Models of electron tomograms showing immunolabelling (red) of endogenous NOGO-B/RTN4B in Huh-7 cells at sheet edges (Ea) and tubules (Eb) of the ER (yellow). NIH/3T3 (**F**, left) and Huh-7 (**F**, right) cell lysates were analysed by Western blotting with NOGO-B/RTN4B antibody to verify the antibody specificity. Bars, 10 μm (**A**,**B**), 1 μm (**C**,**D**), 500 nm (**E**). Scale bars in E are in the perspective view and apply to the centre of the image.

Most of the data concerning the localization of RTN4 isoforms on ER membranes are based on over expression data^4^,^20^,^21^,^22^ although few studies have been done using antibodies against endogenous proteins^23^,^24^,^25^. In our study, we used cell lines with different ER morphology and network organization to analyse the localization of endogenous NOGO-B/RTN4B and NOGO-A/RTN4A. ER in Huh-7 cells consist of highly abundant, large ribosome studded fenestrated sheets along with tubules at cell periphery^26^, whereas the ER in NIH/3T3 cells comprises of large but mostly intact ER sheets at the perinuclear region and abundant tubular network covering most of the cytoplasmic space. In short, ER in Huh-7 cells is mainly composed of sheets and in NIH/3T3 of tubules. Light microscopy (LM) analysis revealed clear co-localization of endogenous NOGO-B/RTN4B with soluble luminal ER marker Hsp47-EGFP in both cell lines (Fig. 2A,B). The overall labelling pattern looked similar in both cell lines, although in Huh-7 cells, the Hsp47-EGFP labelling was most distinctive in the perinuclear region, where ER sheets dominate. In both cell types, co-localization was lowest at perinuclear region, and highest at cell periphery, where ER is mostly in tubular form (insets). Quantitation gave Pearson’s correlation coefficient values 0.6 for NIH/3T3 and 0.5 for Huh-7cells (Supplementary Fig. S1A). Western blotting revealed one clear band corresponding to the size of NOGO-B/RTN4B in Huh-7 and NIH/3T3 cell lysates, verifying the specificity of the antibody (Fig. 2F). To test the specificity of the anti-NOGO-B/RTN4B antibody in immunofluorescence, random fields of silenced and control Huh-7 cells were imaged with the same parameters, and relative mean signal intensities were determined (Supplementary Fig. S1B,C). 60% reduction in relative mean signal intensity matches well with the Western blotting results showing 50–60% reduction of NOGO-B/RTN4B after 48 hours silencing (Supplementary Fig. S3A,B).

Pre-embedding immuno electron microscopy (immuno-EM) verified the ER localization in both cell lines (Fig. 2C,D). In this technique, saponin-permeabilized cells were immunolabelled using1.4 nm-gold particle conjugated Fab fragments as secondary detection step and silver enhanced prior plastic embedding. Because of the nature of the silver enhancement reaction, stereological quantitation approaches are not applicable, however, in addition to revealing the localization, some conclusions on quantities can be drawn based on the cluster size and amounts. The labelling was strongest in cell periphery on ER tubules, although punctate labelling on ER sheets could be found too. The labelling pattern in Huh-7 cells was further analysed at 3D using ET, which allowed more reliable identification of sheets and tubules (Fig. 2E). First, labelling localized clearly on tubules and sheet edges. Secondly, flat parts of ER sheets were devoid of labelling. In addition to sheet edges, membrane curvature could also be found from fenestrations, which comprise both positive and negative curvature. Interestingly, we found that most of the small and regular shaped fenestrations^26^ on ER sheets were devoid of labelling, supporting the suggestion that NOGO-B/RTN4B prefers positively curved membranes. However, occasionally we could find labelling associated to slightly larger and more irregular fenestrations, which were clearly smaller than ER polygons (Supplementary Video S1). We cannot exclude the possibility that these structures might be in a process of forming a polygon opening, or polygon undergoing a closure.

**Figure 3:**
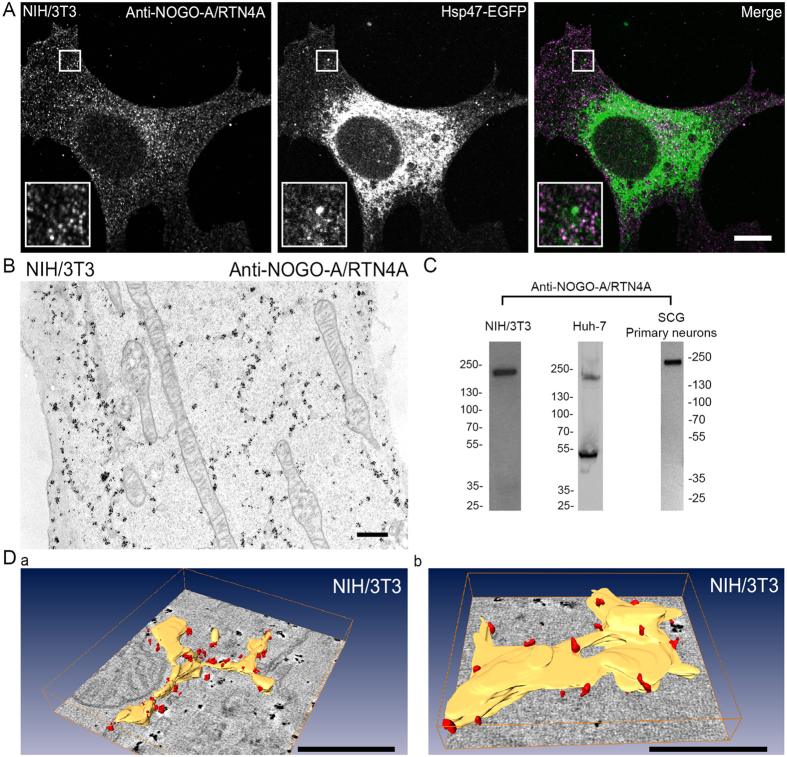
NOGO-A/RTN4A localizes to ER tubules and sheet edges in fibroblast cells. (**A**) Confocal LM images of NIH/3T3 cells showing localization of immunolabelled endogenous NOGO-A/RTN4A and ER marker Hsp47-EGFP. Insets show higher magnification of boxed areas. (**B**) TEM micrograph of NIH/3T3 cell showing immunolabelling of endogenous NOGO-A/RTN4A. NIH/3T3 (**C**, left), Huh-7 (**C**, middle) and primary mouse neuronal (**C**, right) cell lysates were analysed by Western blotting with NOGO-A/RTN4A antibody to verify the antibody specificity. (**D**) Models of electron tomograms showing immunolabelling (red) of endogenous NOGO-A/RTN4A in NIH/3T3 cell at sheet edges and tubules of the ER (yellow). Bars, 10 μm (**A**), 500 nm (**B**,**D**). Scale bars in D are in the perspective view and apply to the centre of the image.

Antibody labelling against endogenous NOGO-A/RTN4A revealed a clear ER network labelling pattern in NIH/3T3 cells (Fig. 3A). Quantitation of endogenous NOGO-A/RTN4A and luminal ER marker labelling gave Pearson’s correlation coefficient value 0.3, which was about half compared to the value between NOGO-B/RTN4B and ER marker in same cells (Supplementary Fig. S1A). Thin section TEM and ET analysis of immunolabelled NIH/3T3 cells confirmed that NOGO-A/RTN4A associated label was on ER tubules and sheet edges (Fig. 3B,D) reminiscent of NOGO-B/RTN4B labelling. NOGO-A/RTN4A localized heavily in the tubular ER close to PM, and, to some extent, also near the PM where no clear ER-connection could be observed (Fig. 3B). We could not detect staining at the extracellular side of the PM, which is in contrast to earlier study showing that NOGO-A/RTN4A localizes to the extracellular side of the PM in fibroblasts^27^. Western blotting revealed one clear band corresponding to the size of NOGO-A/RTN4A and no band in the area corresponding to the smaller size of NOGO-B/RTN4B in NIH/3T3 cell lysates, verifying the specificity of this antibody, too (Fig. 3C). In Huh-7 cells the same antibody gave two bands corresponding to the size of NOGO-A/RTN4A and NOGO-B/RTN4B, suggesting that although antibody is specific for mouse proteins, it may have some cross-reactivity in human cell types (Fig. 3C). Antibody specificities in NIH/3T3 cells allowed simultaneous immunofluorescence labelling of endogenous NOGO-A/RTN4A and NOGO-B/RTN4B (Supplementary Fig. S2). Results confirmed the localization of these two proteins on ER. However, in the merged image, green and magenta fluorescent signals alternated along the ER profiles rather than showing a homogeneous overlap (in white). Pearson’s correlation coefficient value was 0.5.

**Figure 4:**
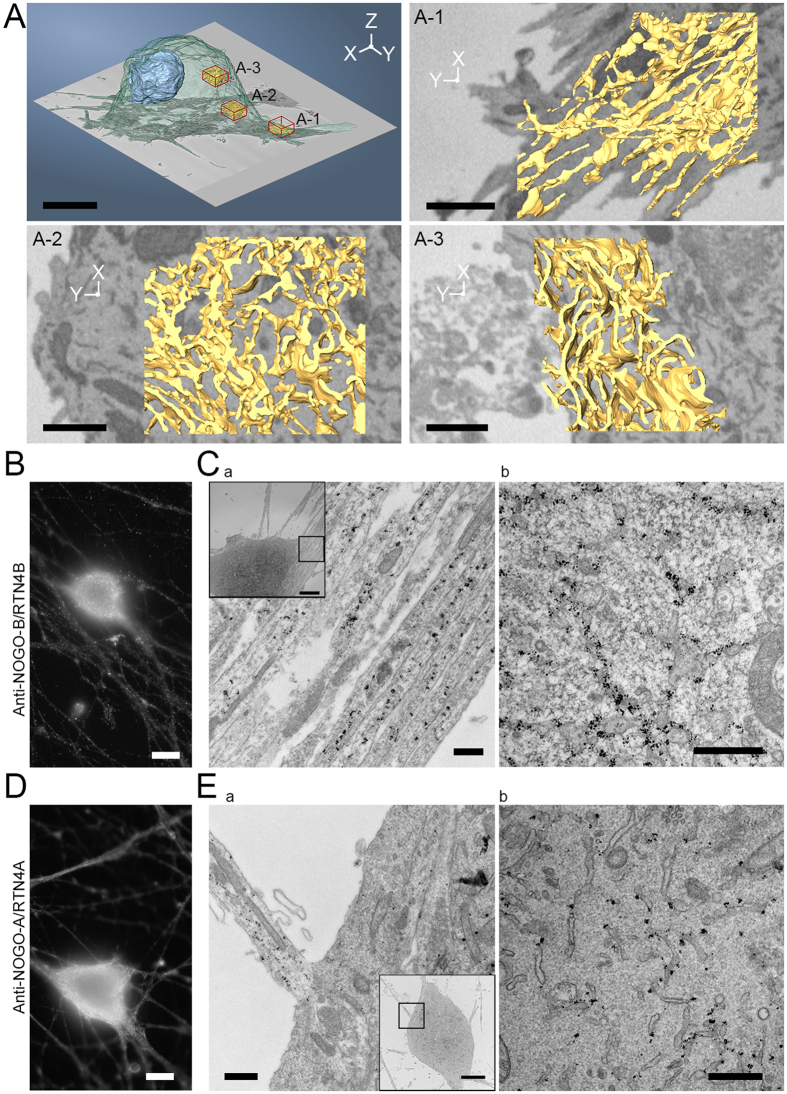
NOGO-B/RTN4B and NOGO-A/RTN4A localize to ER tubules and sheet edges in primary mouse neurons. (**A**) A model of SCG primary mouse neuron segmented from SB-EM data showing outlines of the cell (transparent green), nucleus (blue), and ER (yellow) at three different regions (red cubes). ER at neurite outgrowths (A-1) was mostly in tubular form while in the pericentriolar area (A-2) the ER comprised of a mixture of tubules and smaller sheets and the area immediately under the PM (A-3) had densely packed large ER sheets. Also see Supplementary Video S2. SCG-primary neurons showing endogenous NOGO-B/RTN4B immunolabelling in (**B**) wide field LM image and (**C**) TEM micrograph at neurite outgrowths (Ca) along with tubules and sheet edges at the cell soma (Cb). SCG-primary neurons showing endogenous NOGO-A/RTN4A immunolabelling in (**D**) wide field LM image and (**E**) TEM micrograph, at neurite outgrowths (Ea) along with tubules and sheet edges under the PM (Eb). Bars, 10 μm (**A**,**B**,**D**), 1 μm (A-1, A-2 and A-3), 500 nm (Ca, Cb, Ea and Eb), 5 μm (insets Ca, Cb, Ea and Eb).

Next, we studied the localization of endogenous NOGO-B/RTN4B and NOGO-A/RTN4A in mouse superior cervical ganglia (SCG) primary neurons. The morphological analysis revealed distinct ER network organizations in various parts of the cell (Fig. 4A; Supplementary Video S2): Immediately under the PM, a layer of densely packed large intact sheets were found (Fig. 4A-3), and towards the pericentriolar area the ER comprised of tubules and smaller sheets (Fig. 4A-2), whereas in neurite outgrowths ER was mainly tubular (Fig. 4A-1). At LM level, labelling of NOGO-B/RTN4B (Fig. 4B) and NOGO-A/RTN4A (Fig. 4D) looked similar, and were found extending from pericentriolar part of the cell to the cell periphery in cell soma as well as in neurites. Immuno-EM of NOGO-B/RTN4B revealed that endogenous protein localized mainly to ER at cell soma (Fig. 4Cb), as well as on ER tubules within neurites (Fig. 4Ca). Similarly as with fibroblasts, some NOGO-A/RTN4A associated label was found at the cytosolic side of the PM without clear presence of ER (Fig. 4Eb) in addition to prominent labelling pattern on ER tubules in cell soma and neurites (Fig. 4Ea). Previously NOGO-A/RTN4A has been reported to localize on the extracellular side of the PM on Dorsal Root Ganglion neurons and myoblasts^27^. Our immuno-EM studies could not verify these findings as no labelling was observed on the extracellular side of the PM. Together, these results indicate that NOGO-A/RTN4A and NOGO-B/RTN4B both localize prominently on ER tubules and sheet edges.

### Manipulation of reticulon 4B levels induces severe morphological changes on ER and overall cell shape

**Figure 5:**
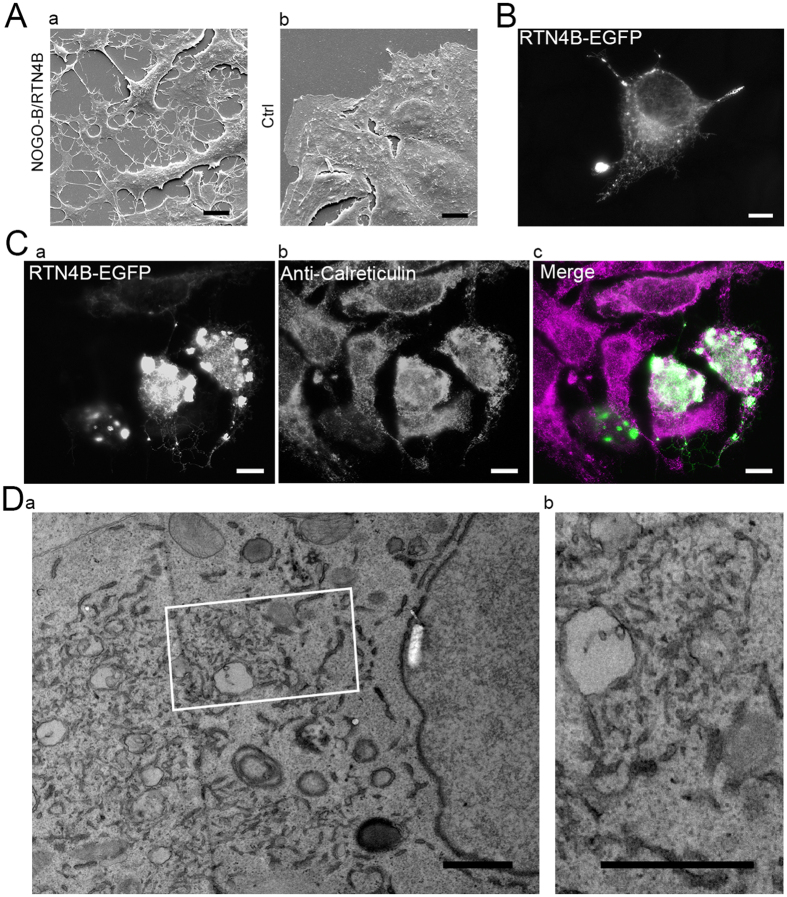
NOGO-B/RTN4B overexpression affects overall cell shape and ER morphology. (**A**) Scanning EM images of Huh-7 cells overexpressing (Aa) pCMV-RTN4B or (Ab) CMVTag1 (control) for 24 hours. (**B**) Wide field LM image of Huh-7 cells overexpressing RTN4B-EGFP for 24 hours showing filopodia like protrusions. Also see Supplementary Video S3. (**C**) Wide field LM image Huh-7 cells overexpressing RTN4B-EGFP (Ca) for 24 hours showing tubular ER network and globular profiles positive for ER marker calreticulin (Cb). (**D**) TEM micrographs of Huh-7 cell co-expressing ssHRP-KDEL and RTN4B-EGFP for 24 hours. (Db) shows a higher magnification of the boxed area in Da revealing a dense network of short ribosome-free tubules connected to ER sheets. Bars, 10 μm (**A**,**B**,**C**), 1 μm (Da and Db).

Overexpression of RTN4B-EGFP in Huh-7 cells induced changes on ER morphology as well as in the overall cell morphology. Scanning EM revealed long filopodia-like protrusions and overall rounding of the cells in response to RTN4B-EGFP expression (Fig. 5A). LM analysis revealed formation of long unbranched ER tubules extending through the cell at moderate level of expression (Fig. 5B). Live cell imaging revealed that these long ER tubules were non-motile in comparison to cells expressing Hsp47-EGFP (Supplementary Video S3). At higher expression levels, formation of globular profiles, positive for endogenous ER protein calreticulin, at varying sizes could also be observed (Fig. 5C). TEM analysis showed that the globular structures observed at LM comprised a densely branched network of thin and rather short tubules connected to more typical ER profiles (Fig. 5D).

**Figure 6:**
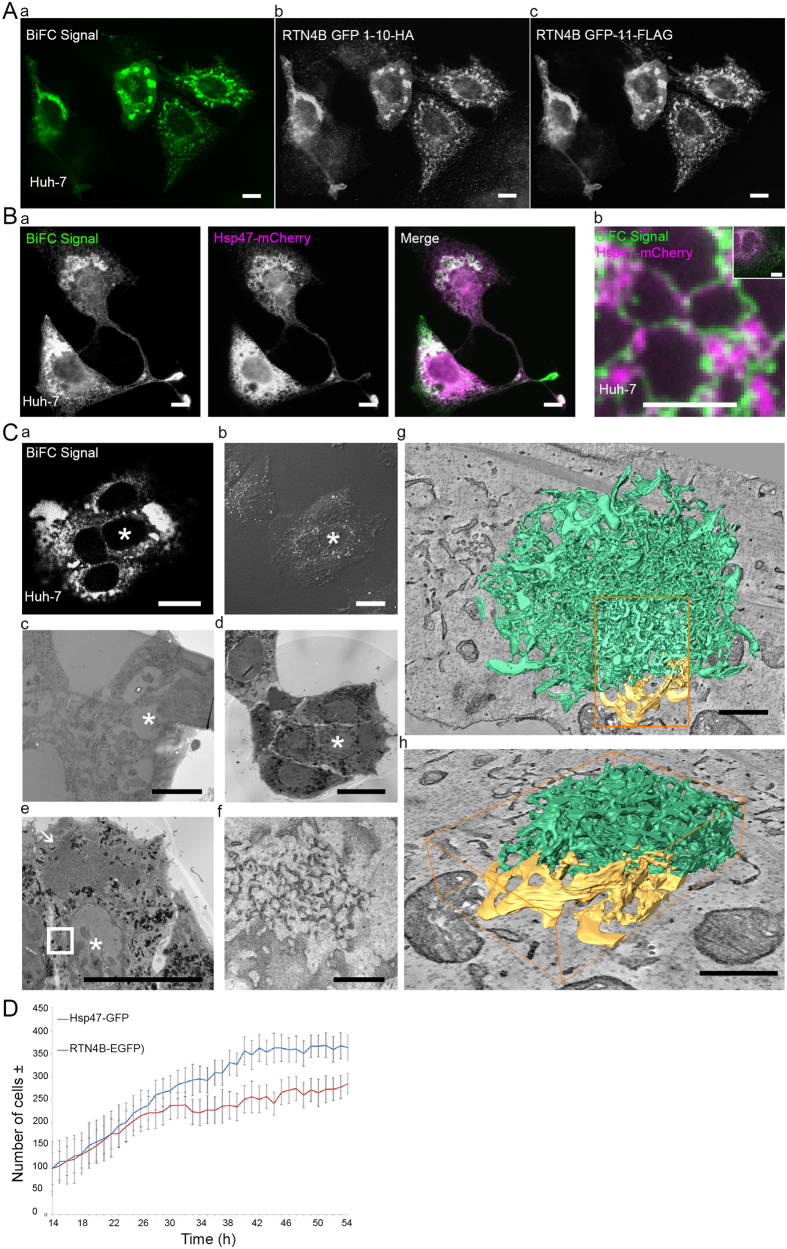
Overexpression of locked NOGO-B/RTN4B dimers induces strong ER tubulation and overexpression NOGO-B/RTN4B hampers cell growth. (Aa) Wide field LM image showing a positive BiFC signal arising from co-expression of RTN4B-GFP1-10-HA and RTN4B-GFP-11-FLAG constructs in Huh-7 cells for 24 hours. Wide field LM images of immunolabelled (Ab) HA and (Ac) FLAG tags verifying the equal expression of both constructs in the cells. BiFC signal (Ba) co-localizes with the ER marker Hsp47-mCherry at tubular ER network and large globular BiFC signal-positive structures in highly over-expressing cells, and (Bb) shows only partial co-localization in moderately over-expressing cells. Inset in Bb showing the overview of the cell. (**C**) CLEM workflow of Huh-7 cells co-expressing RTN4B-GFP1-10-HA and RTN4B-GFP-11-FLAG. (Ca) Confocal LM image showing the BiFC signal, (Cb) phase contrast LM image and low magnification TEM micrographs from consecutive (Cc) 60 nm and (Cd) 250 nm thin sections from the same cell cluster. (Ce) TEM micrograph of the whole cell depicted with asterisk in Ca-Cd and (Cf) the higher magnification micrograph of a small globular ER cluster depicted by a white box in Ce. Arrow in Ce depicts another, much larger globular ER cluster in the same cell. (Cg,Ch) 3D- models generated from serial TEM tomograms from the same boxed area in Ce revealing that normal fenestrated sheets (yellow) are directly connected to heavily tubular smooth ER (green). Cg shows the whole globular ER cluster, and the magnified part of the model shown in Cf is depicted by an orange box. (**D**) Cell-IQ data showing number of cells overexpressing RTN4B-EGFP (red line) vs control (Hsp47-EGFP) (blue line). Imaging started 14 hours after transfection. Also see Supplementary Video S4. Bars, 10 μm (A,Ba and inset Bb), 2.5 μm (Bb), 20 μm (Ca, Cb, Cc, Cd and Ce), 500 nm (Cf, Cg and Ch).

It has been shown before that NOGO-B/RTN4B forms dimers/oligomers besides interacting with other proteins^10^. We used bimolecular fluorescence complementation assay (BiFC)^28^ to lock NOGO-B/RTN4B homo-oligomers to study their impact on ER structure. For this, we constructed NOGO-B/RTN4B tagged with GFP1-10 or GFP11 at the C- terminus of the protein. A clear BiFC signal was obtained in Huh-7 cells co-expressing these truncated GFP constructs (Fig. 6Aa) confirming the close interaction between RTN4Bs. In highly overexpressing cells, the signal came from tubular network and large bright globular structures throughout the network, which colocalized with soluble luminal ER marker Hsp47-mCherry (Fig. 6Ba). In mildly over-expressing cells the BiFC signal originated from the tubules and sheet edges in Huh-7 cells (Fig. 6Bb), verifying that the functional BiFC NOGO-B/RTN4B GFP-constructs localized as the endogenous reticulon 4B protein (Fig. 2). Locking of NOGO-B/RTN4B oligomers induced similar filopodia-like protrusions as the over-expression of NOGO-B/RTN4B-EGFP (see Fig. 5B). Correlative light and 3D- electron microscopy (CLEM) analysis revealed that these globular BiFC-positive structures were composed of a dense network of short ribosome-free tubules that were connected to sheets at the immediate vicinity of the cluster (Fig. 6C; Supplementary Video S4). These structures were reminiscent of tight tubular ER clusters that has been shown to form upon ATP depletion^29^. Similar tubular ER structures could be found also from cells overexpressing NOGO-B/RTN4B-EGFP but mostly after longer expression times indicating that these structures are not specific consequence of locked oligomers but rather that locked oligomers are more prone to induce such structures.

To study the role of NOGO-B/RTN4B on the cell growth rate, we subjected Huh-7 cells over-expressing NOGO-B/RTN4B-EGFP or as a control Hsp47-EGFP, to time-lapse video microscopy (using continuous cell culturing and imaging platform Cell-IQ) and collected images at 1 h intervals between t_14 h_–t_48 h_ post transfection. The analysis revealed that cells expressing Hsp47-EGFP or NOGO-B/RTN4B-EGFP showed similar growth pattern for the first 24h post transfection, after which the growth rate of NOGO-B/RTN4B-EGFP overexpressing cells started to decline (Fig. 6D). The control cells continued similar growth pattern for additional 17 h, after which the growth rate reached a plateau, most likely due to reduced nutrients in the growth media.

**Figure 7:**
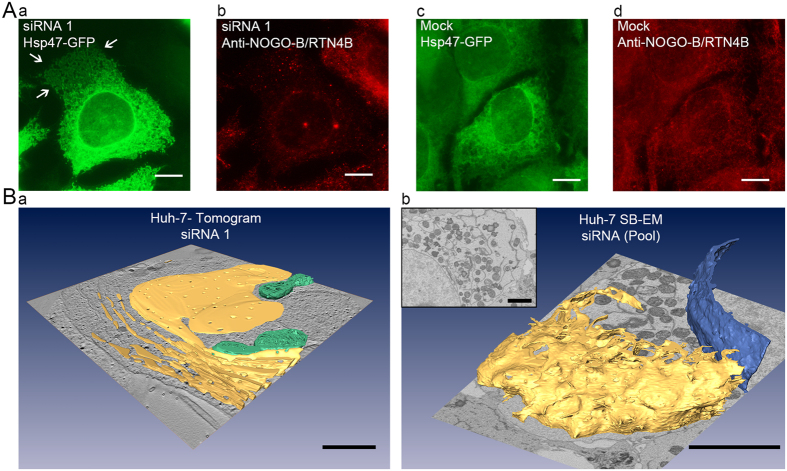
RTN4 depletion causes the loss of ER tubules and generation of large ER sheets that can become stacked in Huh-7 cells. (**A**) Wide field LM images showing RTN4 depleted cell with larger peripheral ER sheets (Aa, Ab; indicated by white arrows) as compared to mock (scrambled siRNA) (Ac, Ad). (**B**) ER (yellow) models from RTN4B depleted Huh-7 cells generated from ET (Ba) and SB-EM (Bb) datasets reveal large stacked sheets. Mitochondria (in Ba) and nuclear envelope (in Bb) are depicted in green and blue, respectively. Also see Supplementary Video S5 (ET) and Supplementary Video S6 (SB-EM). Bars, 10 μm (**A**), 1 μm (Ba and inset of Bb), 5 μm (Bb).

Because of the sequence similarity between RTN4 isoforms, isoform-specific siRNAs would have been very challenging to design and instead, siRNAs recognizing all *RTN4* isoforms were used (Fig. 7). According to Western blotting a pool of 3 constructs or just one construct lead to 50–60% reduction of NOGO-B/RTN4B after 48 hours (Supplementary Fig. S3A,B). LM analysis of RTN4-depleted cells revealed an increased number of peripheral ER sheets (Fig. 7Aa,b) compared to scrambled siRNA (Fig. 7Ac,d). These findings were supported by 3D-EM (Fig. 7B). ET revealed that RTN4-depleted Huh-7 cells contained extended ER sheets, which in extreme cases were stacked at the cell periphery. The fenestrations typical for ER in these cells^30^ could be seen in the stacked ER sheets (Fig. 7Ba). SB-EM revealed large ER sheets through the cytoplasmic space (Fig. 7Bb). Nucleus, mitochondria and Golgi morphology appeared normal. Thin section TEM analysis confirmed that similar morphological changes were produced with each silencing construct separately and with a pool of 3, thus ruling out any off-target effects (Supplementary Fig. S3C).

## Discussion

Reticulons have been shown to have a role in many cellular functions ranging from inhibition of neural outgrowth and axonal regeneration in the central nervous system^3^ to ER morphogenesis^4^ and ER stress-induced apoptosis^31^,^32^. Recently, additional roles, including involvement in STIM1-Orai1-Coupling and Store-operated Calcium Entry^23^, autophagy^33^, DNA binding^34^, and inflammatory-related functions^35^, have been reported for RTNs, and their involvement in neurodegenerative diseases such as Alzheimer’s disease, amyotrophic lateral sclerosis, multiple sclerosis, as well as hereditary spastic paraplegia^36^, are evident. In addition to conflicting results reported from neurobiology and cell biology regarding the localization and expression of NOGO-A/RTN4A, a clear and detailed description of the expression, localization and functional significance of the other reticulon 4 isoforms has been lacking. Here we report, based on transcriptome analysis, that RTN1-4 are simultaneously expressed, at different levels, in cultured hepatoma cells. Consistent with previous results^36^, our qPCR expression profiling of *RTN4* isoforms detected 5 isoforms in cultured hepatoma, fibroblast and primary neuronal cells: A-D isoforms were expressed in varying degrees and E was present at relatively low level. While RTNs have been suggested to have general roles on ER, the simultaneous presence of *NOGO-B/RTN4B* and *NOGO-A/RTN4A*, in addition to other isoforms, in all three cell lines suggests distinct cellular roles for these proteins.

It has been suggested that the hydrophilic loop of the NOGO-A/RTN4A localizes to the extracellular side of the PM and binds to its axonal receptor^37^. Controversially, based on several cell biological studies, RTNs localize to the highly curved regions of ER^4^. N-terminus of all RTN4 isoforms lack a specific signal sequence for ER translocation, which might account for their reported presence both in the ER and at the extracellular side of the PM^5^,^6^. Here we addressed the discrepancy related to the localization of the NOGO-A/RTN4A by detecting endogenous NOGO-A/RTN4A in fibroblasts and neurons. Majority of the labelling was detected on the cytoplasmic side of the cells, at ER tubules and sheet edges, while the extracellular side of the PM was mostly void of the labelling. We also observed abundant labelling at cell periphery next to the PM either in conjunction with ER profiles or without an immediate connection to ER. Based on theoretical models, several RHD conformations are possible and the RTNs may be able to insert and flip–flop within PM, giving rise to several alternative orientations of the proteins^4^,^5^,^38^,^39^,^40^. The detected labelling at cell periphery might originate from PM or from ER profiles located above or under the thin section. The different membrane topology at highly curved ER areas or under the flat PM suggests that NOGO-A/RTN4A performs divergent functions at different cellular locations, which might be explained by *e*.*g*. the interacting protein environment, indicating that NOGO-A/RTN4A might have additional roles beyond ER morphogenesis. The controversial results obtained from cell- and neurobiology might be explained by the different functions these cells perform.

RTNs have also been suggested to form arc-shaped oligomers^10^, which would generate and stabilize membrane curvature by wedging and scaffolding mechanisms. Structural evidence for the proposal has so far been missing. Here we show by immuno-EM and -ET that the endogenous NOGO-B/RTN4B and NOGO-A/RTN4A localize to curved ER membranes in distinct patterns. NOGO-B/RTN4B and NOGO-A/RTN4A localized to curved ER membranes, *i.e.* tubules and sheets edges and while it was devoid from small sheet fenestrations, labelling was occasionally seen at larger fenestrations. Clusters of label were seen at relatively regular intervals covering the tubule length and sheet edges indicating that endogenous NOGO-B/RTN4B and NOGO-A/RTN4A do not cover the whole tubule length but rather are present at distinct regions along the structure. Interestingly, the labelling pattern at ER tubules, sheet edges and adjacent to PM were slightly different; while the cluster size was relatively large at ER tubules, fewer and smaller silver/gold clusters were seen at sheet edges and under PM. This might indicate that NOGO-B/RTN4B and NOGO-A/RTN4A forms oligomers at distinct spots at ER tubules and through oligomerization bends the ER membranes, whereas smaller oligomers, or singular proteins, could be residing at sheet edges and at PM.

Artificial over-expression of NOGO-B/RTN4B induced proliferation and immobilization of ER tubules along with long filopodia-like protrusions and rounding of the cell. In agreement with previous results showing that manipulation of RTN4C levels inhibit cell growth^41^, these events induced by over-expression of NOGO-B/RTN4B were conveyed into a significant drop in the cell growth rate and eventually led to cell apoptosis. The altered ER morphology upon NOGO-B/RTN4B over-expression does not resemble the organized smooth ER structures that have been described after over-expression of several ER membrane proteins^42^, but rather the random tubular SER patches induced by a treatment with the drug 1-Phenyl-2-decanoyl-amino-3-morpholino-1-propanol (PDMP)^43^. Finally, in the light of these results, using the NOGO-B/RTN4B overexpression constructs as general markers for ER tubules should be carefully considered as even small changes in protein levels affect sheet-tubule balance and thereby induce clear changes in the ER morphology. Recent development in targeted genome editing technology^44^,^45^ provides solution as this approach can be used to generate stable cell lines expressing epitopically tagged versions of various ER membrane proteins at endogenous levels to avoid changes in sheet-tubule balance or expansion of the membrane.

## Methods

### Cell culture, constructs, overexpression and silencing

NIH/3T3 (CRL-1658; ATCC, LGC Standards GmbH, Germany) were cultured in DMEM (BioWhittaker, Lonza, Verviers, Belgium) and Huh-7 (JCRB0403; Japanese Collection of Research Bioresources Cell Bank, Osaka, Japan) in EMEM (Lonza), containing 5 or 10% fetal bovine serum (Gibco, Invitrogen, Thermo Fisher Scientific, Waltham, MA) and other supplements (Lonza). Cervical ganglion primary sympathetic neurons from new-born Swiss type NMRI-mouse (SCG primary neurons) were cultivated on polyornitin-laminin (Sigma-Aldrich, St. Louis, MO) coated coverslips in neurobasal-medium containing B27 (Gibco, Invitrogen) and 30 ng/ml mouse 2.5 S nerve growth factor (NGF; Promega, Madison, WI). Mouse E16 (embryo collected 16 days after fertilization) primary cortical neurons were used for the qPCR. pssHRP-KDEL^46^ and pHsp47-EGFP^47^ were provided by the respective laboratories. Construction of Hsp47-mCherry has been described previously^30^. Reticulon 4B gene was amplified from U-2 OS cDNA produced from extracted total mRNA and then cloned into pCMV-Tag1 (Agilent Technologies, Espoo, Finland). EGFP was subcloned into C-terminus of the pCMV-RTN4B expression vector by PCR from pEGFP-N1 (Clontech, Takara Bio Inc., Tokyo, Japan) resulting in pCMV-RTN4B-EGFP. For the BiFC constructs, RTN4B in pENTR221gateway compatible vector (without stop codon) from Orfeome library, GBU, Helsinki University, was taken through L.R reaction (Gateway^®^ Cloning, Life Technologies, Thermo Fisher Scientific) against pDEST-GFP-1-10-N3 or pDEST-GFP-11- N3 gateway compatible vectors (Gift from Maria Vartiainen’s lab, University of Helsinki) creating C-terminal fusion vectors RTN4b-GFP-11 and RTN4B-GFP-1-10. For overexpression studies and all DNA transfections, Fugene HD (Promega) was used according to manufacturer’s instructions and cells fixed for analysis 24 h or 48 h post-transfection. For co-transfections of two and three plasmids a ratio of 1:2 (wt:wt) and 0.5:0.5:4 (wt:wt:wt) was used. RTN4 silencing was done using INTERFERin (Polyplus-transfection, France) for 48 h according to manufacturer’s instruction, and the ER marker was transfected 24 h post silencing for the remaining 24 h. For silencing single or total of pooled target-specific siRNA oligonucleotides from Thermo Scientific Dharmacon (Lafayette, CO) (sequences: 5′-GCGCAAAGCUGAAUGAAAAUU-3′, 5′-GUUCAGAAGUACAGUAAUUUU-3′ and 5′-CGGUAAAGCAGGAAUGACAUU-3′, the 2-nucleotide overhanging uridine is indicated as UU)^48^ were used. The silencing efficiency of the siRNAs was quantified using Western blotting.

### Antibodies

Antibodies against reticulon 4B (AB-163; Kinasource Ltd, Dundee, UK), reticulon 4A (ab62024; Abcam, Cambridge, UK), HA (MMS-101R-50; Covance, Princeton, NJ), FLAG (F7425; Sigma-Aldrich), calreticulin (2679S; Cell Signalling Technologies, MA) and β-actin (ab8227-50; Abcam) were used as primary antibodies. When indicated, rabbit anti-sheep bridging antibody (313-001-003; Jackson ImmunoResearch Labs Inc., West Grove, PA) was used. Secondary antibodies were Rhodamine Red-X (016-290-084; Jackson ImmunoResearch), Alexa 647 (A31571; Life technologies), Alexa 488 (A-11008; Life Technologies) and 1.4 nm nanogold-conjugated anti-rabbit antibody (Nanoprobes, Stony Brook, NY).

### Immunofluorescence staining and Western blotting

For NOGO-B/RTN4B labelling, cells were fixed with −20 °C methanol, blocked with 10% goat serum (Gibco-Life Technologies) and 1% BSA (bovine serum albumin), labelled with indicated antibodies and mounted in Mowiol (Hoechst, Frankfurt, Germany) supplemented with Dabco (Sigma-Aldrich). All the other samples were fixed with 4% formaldehyde (Electron Microscopy Sciences, Hatfield, PA), 0.1 mM MgCl_2_, and 0.1 mM CaCl_2_ in phosphate-buffered saline (PBS), quenched with 50 mM NH_4_Cl, permeabilized with 0.1% Triton X-100, and then blocked with 0.2% BSA in Dulbecco PBS. When appropriate, the cells were then incubated consecutively with primary and secondary antibodies, diluted in blocking solution. Samples were mounted in Mowiol supplemented with Dabco. Western blotting was done with indicated antibodies according to manufacturer’s instructions and by using standard protocols.

### Light microscopy and image quantitation

Wide-field images of fixed cells were taken with Zeiss AxioImager M2 482 epifluorescence microscope equipped with 63 × /Plan-Apochromat/1.40 oil/M27 and 483 AxioCam HRm camera (Zeiss, Oberkochen, Germany) (Figs 4,5 and 6A,B), Leica DM6000B upright fluorescence wide field microscope equipped with 40X/1.25-0.75 HCX PL APO CS oil objective, Hamamatsu Orca-Flash4.0 V2 sCMOS camera (Wetzlar, Germany) (Supplementary Figure S1) or with Olympus BX61 wide field microscope (Tokyo, Japan) (Fig. 7). Images were acquired with AxioVision4 (Zeiss), LAS X software (Leica) or Fluoview software (Olympus). Confocal images of fixed cells were taken with LSM880 confocal laser scanning microscope (Zeiss) with a 63X plan-apochromat (NA = 1.40) oil objective, GaAsP detector and ZEN 2 software (Zeiss) (Figs 2 and 3) or with inverted TCS SP5II HCS A confocal (Leica, Mannheim, Germany) using an HCX PL APO (Leica) lambda blue 63.0 × /1.2 W Corr/0.17 CS objective, HyD 4 detector (Leica) with either standard or BrightR detection mode, Scan DIC and blue (Ar 488 nm/35 mW) laser line, and RSP 500 beam splitter (Fig. 6Ca). Live Huh-7 cells were imaged at 37 °C and 5% CO_2_ on glass-bottom dishes (MatTek, Ashland, MA). Videos were acquired using a PLANAPO 60 × 1.45 (oil) objective with additional 1.6× magnification in an IX-71 inverted microscope (Olympus) equipped with a TILL imaging system (TILL Photonics, FEI Company, Hillsboro, OR), iXon camera (Andor, Oxford Instruments, Abingdon, UK). Pearson’s correlation coefficiencies were calculated using Microscopy Image Browser (MIB)^49^ for square ROIs placed on the cell lamella (n > 10). As a negative control, the Pearson’s correlation coefficient was also calculated for the same ROIs after rotating one of the two channels by 90°^49^. Cell segmentation and mean intensity measurements of NOGO-B/RTN4B in mock and silenced Huh-7 cells (n = 20) were done using MIB.

### Electron microscopy

Cells grown on glass coverslips were cytochemically stained and flat embedded as described previously^50^. Cells were fixed with 2% glutaraldehyde (Sigma-Aldrich) and 1.5% formaldehyde in 0.1 M sodium cacodylate buffer, pH 7.4, for 20 min at room temperature (RT). Cytochemical staining with 3,3′-diaminobenzidine (TAAB, Berks, UK) was done for pssHRP-KDEL transfected cells. Cells were then postfixed with 1% reduced osmium tetroxide in sodium cacodylate buffer for 1 h on ice, dehydrated through series of ethanols and acetone, and infiltrated with Epon (TAAB 812) for 2 h prior 14- h- polymerization at 60 °C. 60-nm or 100-nm thin sections were cut, post-stained with uranyl acetate and lead citrate and imaged with Tecnai 12 (FEI Company, at 80 kV or 120 kV equipped with Orius SC 1000B (Gatan Inc.) CCD camera. For immuno-EM the cells were fixed with paraformaldehyde-lysine-periodate -fixative^51^. Cells were permeabilized with 0.01% saponin (Sigma-Aldrich) and immunolabelled with anti-NOGO-B/RTN4B followed with rabbit anti-sheep bridging antibody or with anti NOGO-A/RTN4A antibody, and 1.4 nm nanogold-conjugated anti-rabbit secondary antibody, silver enhanced with HQ Silver kit (Nanoprobes, Stony Brook, NY) and gold toned with 0.05% gold chloride. Finally cells were processed for osmication, dehydration, Epon embedding, sectioning and imaging as described above.

### Scanning electron microscopy and SB-EM

For scanning electron microscopy (SEM), cell monolayers overexpressing pCMV-RTN4B-HA or pCMVTag1 for 24 h were grown on glass coverslips and fixed with 2% glutaraldehyde in 0.1 M sodium cacodylate buffer, pH 7.4 for 30 min at RT and post-fixed with 1% osmium tetroxide in 0.1 M sodium cacodylate buffer for 60 min at RT. The samples were dehydrated through series of ethanols, followed by overnight dehydration with hexamethyldisilazane (Sigma-Aldrich). All the samples were coated with platinum using Agar sputter coater (Agar scientific Ltd, Essex, UK). Imaging was done using Zeiss DSM 962 (Oberkochen, Germany) at 10 kV. SB-EM samples of mouse primary neurons and RTN4 knockdown Huh-7 cells were prepared as described previously^26^. Images were acquired with a FEG-SEM Quanta 250 (FEI Company, Hillsboro, OR) equipped with a microtome (3View; Gatan Inc., Pleasanton, CA), using a backscattered electron detector (Gatan Inc.). The imaging was done using 0.3-Torr pressure, 2.5 kV beam voltage and spot size 3. Image processing and segmentation were done using MIB. Visualization of models and rendering of videos were done in Amira (VSG, FEI Company).

### Electron tomography

ET was done on serial 250-nm thick sections as previously described^52^, except that the tilt series images between ±62° were acquired with an UltraScan 4000 CCD camera, 4 k × 4 k (Gatan Inc.) at nominal magnification of 6.500×, 7.800× or 9.600×. Dual axis tilt series were acquired using SerialEM software running on a Tecnai FEG 20 microscope (FEI Company, Hillsboro, OR) operating at 200 kV. Gold particles on tomograms were quantified by manually tagging on separately modelled ER structures. Reconstructions were done using IMOD software^53^ followed by visualization and modelling using MIB and Amira (VSG, FEI Company). The presented 3D models of ER are shown in perspective view, where the bars apply to the centre point of the image except in Fig. 6Cf.

### Expression profiling SOLiD sequencing and qPCR

Three separate total RNAs were extracted from Huh-7 cells with TRIzol (Invitrogen) reagent according to manufacturer’s protocol. Messenger RNA was further extracted from the samples using mRNA isolation kit (Roche, Basel, Switzerland). cDNA libraries from 100 ng of isolated mRNAs were produced using Ovation^®^ RNA-Seq System V2 kit (NuGEN, Leek, The Netherlands). Samples were barcoded and thereafter sequenced using a paired end mode with SOLiD 5500XL (Life Techologies). The SOLiD pair-end sequencing reads were mapped to human transcriptome using Tophat^18^ with RefSeq gene annotation from UCSC (University of California, Santa Cruz, CA) genome browser. The expression level of transcripts was estimated by Cufflinks^19^. Aligned read data from SOLiD sequencing is stored in European Nucleotide Archive (ENA, accession PRJEB12539). The total mRNA from Huh-7, NIH/3T3 and primary mouse (E16) cortical neuronal cells was extracted as mentioned above and reverse transcribed using Roche Transcriptor First Strand cDNA Synthesis Kit. qPCR was performed using iQ™ SYBR^®^ Green Supermix (Bio-Rad Laboratories, Inc, Hercules, CA) with CFX384 Touch system (Biorad). Four technical replicates were used for every isoform. Primers used for the qPCR were provided by Oligomer Oy (Helsinki, Finland) and are presented in Supplementary Table S1. Results are normalized for NOGO-B/RTN4B and expressed as the mean ± SE, where housekeeping human or mouse genes β-actin were used. Results were assessed using 2 tailed student *t* test. *P* < *0*.*05* were considered statistically significant.

### Cell proliferation

Huh-7 cells were grown on 24-well plates and transfected with either RTN4B-EGFP or with control (ER marker Hsp47-EGFP). The cells were grown on a continuous cell culturing platform with integrated optics for phase contrast imaging and machine vision technology (Cell-IQ, CM Technologies Ltd, Tampere, Finland) in a humidified, 5% CO_2_ atmosphere at 37 °C for approximately 72 h post transfection. Four random positions from each of the three replicates were imaged at 1 h intervals. The total cell count was measured from 14 h to 72 h post transfection using MIB. Cells were recognized and automatically counted by segmenting fluorescent images in MIB. Graphs of averaged cell numbers were generated, normalized and statistically analysed in Excel (Microsoft).

### Statistical testing

Statistical analysis of cell growth rates of Huh-7/Hsp47-EGFP (control; average n/h = 324 cells) compared to Huh-7/RTN4B-EGFP (NOGO-B/RTN4B overexpression; n/h = 355 cells) were done to data derived from 4 parallel samples and 3 replicates. Degrees of freedom (d.f._control_ = 14; d.f_NOGO-B/RTN4B_ = 21) were calculated with Welch-Satterthwaite equation. Statistical significance of normally distributed data was calculated with Student’s *t*-test (Excel, Microsoft), assuming unequal variances. The *p*-values were tested for time points 14/24 h (p > 0.1) and 42/53 h (p < 0.025).

## Additional Information

**How to cite this article**: Rämö, O. *et al*. NOGO-A/RTN4A and NOGO-B/RTN4B are simultaneously expressed in epithelial, fibroblast and neuronal cells and maintain ER morphology. *Sci. Rep.* **6**, 35969; doi: 10.1038/srep35969 (2016).

**Publisher’s note:** Springer Nature remains neutral with regard to jurisdictional claims in published maps and institutional affiliations.

## Supplementary Material

Supplementary Information

Supplementary Video S1

Supplementary Video S2

Supplementary Video S3

Supplementary Video S4

Supplementary Video S5

Supplementary Video S6
