# Supplementary material for: NOGO-A/RTN4A and NOGO-B/RTN4B are simultaneously expressed in epithelial, fibroblast and neuronal cells and maintain ER morphology
Source: Sci Rep. 2016 Oct 27;6:35969. doi: 10.1038/srep35969 (PMC5081510; doi:10.1038/srep35969)
Supplement: Supplementary Information [file srep35969-s1.pdf]

SREP-16-11884B Supplementary information

NOGO-A/RTN4A and NOGO-B/RTN4B are simultaneously expressed in epithelial, fibroblast and neuronal cells and maintain ER morphology

Olli Rämö[a*], Darshan Kumar[a*], Erika Gucciardo[a], Merja Joensuu[a], Maiju Saarekas[a], Helena Vihinen[a,b], Ilya Belevich[a,b], Olli-Pekka Smolander[c], Kui Qian[c], Petri Auvinen[c] and Eija Jokitalo[a,b]

[a]Cell and Molecular Biology Program, [b]Electron Microscopy Unit, and [c]DNA Sequencing and Genomics Laboratory, Institute of Biotechnology, University of Helsinki, Helsinki, Finland

*These authors contributed equally to this work

Address correspondence to: Eija Jokitalo (Eija.Jokitalo@Helsinki.fi)

Supplementary Figures

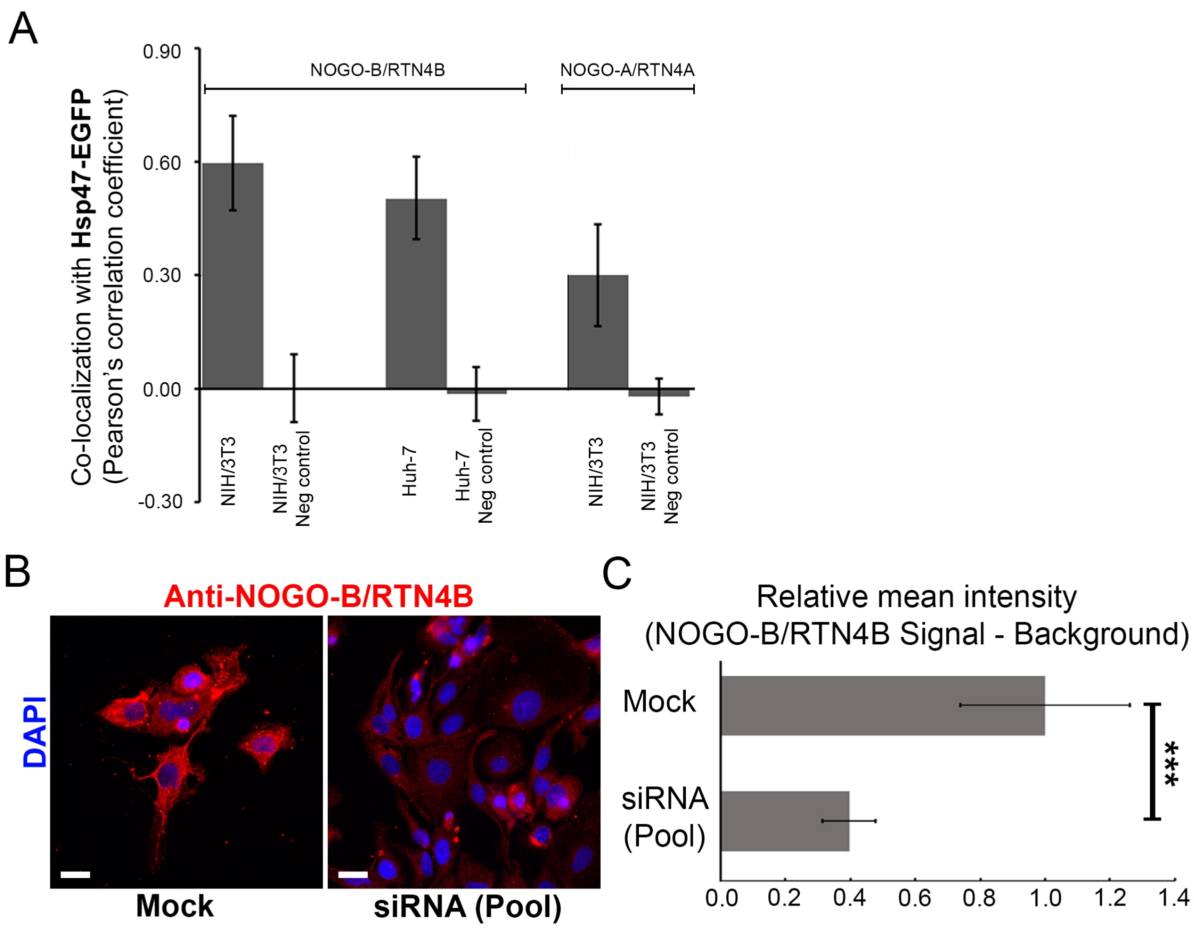

Supplementary Figure S1. (A) NOGO-B/RTN4B and NOGO-A/RTN4A co-localize with the ER marker Hsp47-EGFP. Co-localization between Hsp47-EGFP and endogenous NOGO-B/RTN4B or NOGO-A/RTN4A were quantified using Pearson's correlation coefficient. For negative control (Neg) one of the channels from the same ROI was rotated by 90°**.** (B) Wide field LM images of mock and RTN4 silenced (pool of 3 siRNAs) Huh-7 cells labelled for endogenous NOGO-B/RTN4B (red) and nucleus (blue). Random fields were imaged with the same imaging parameters and relative mean intensities of NOGO-B/RTN4B fluorescent signal was determined (C). Bars, 30 µm.

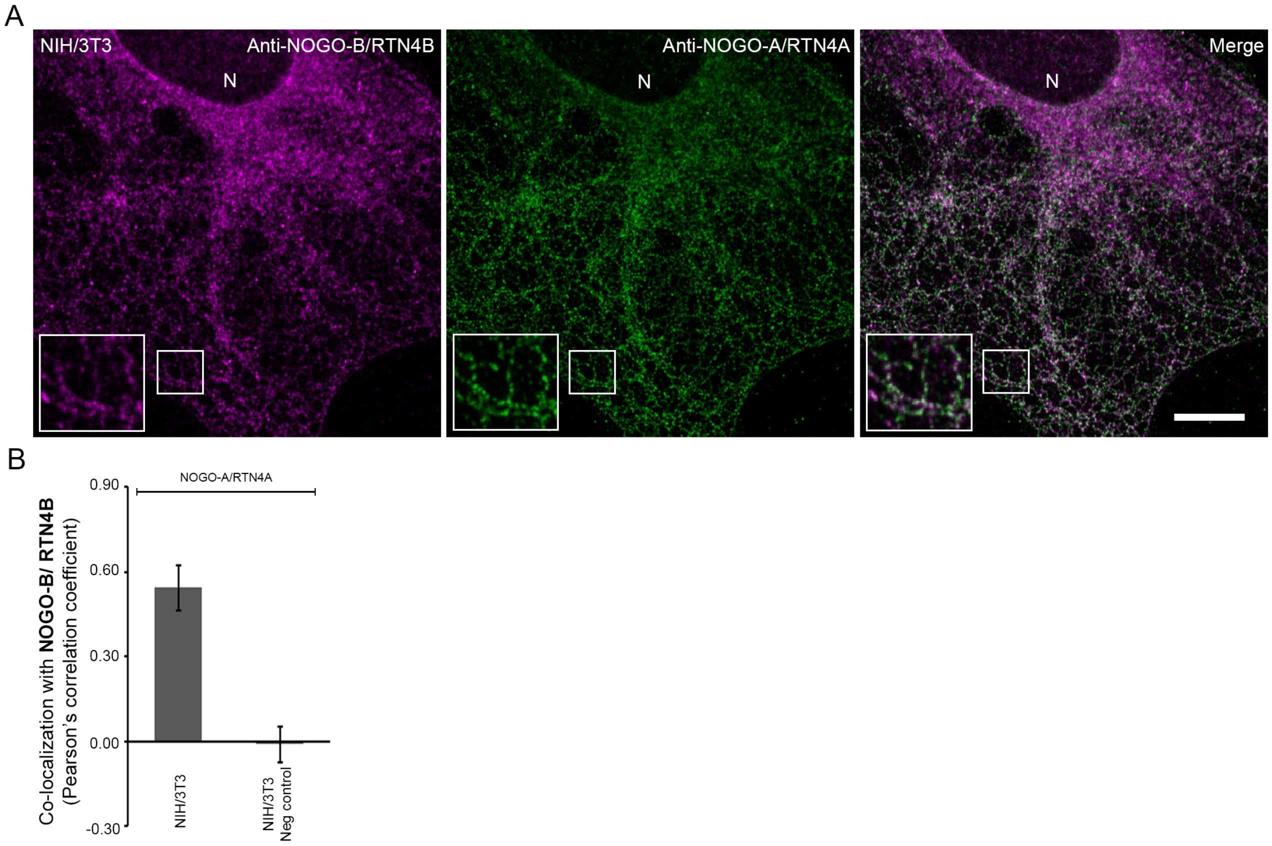

Supplementary Figure S2. NOGO-B/RTN4B co-localizes with NOGO-A/RTN4A in NIH/3T3 cells. (A) Confocal LM images of NIH/3T3 cells showing localization of immunolabelled endogenous NOGO-B/RTN4B and NOGO-A/RTN4A. Insets show higher magnification of boxed areas. Nucleus of the cell is depicted with N. (B) Co-localization between endogenous NOGO-

B/RTN4B and NOGO-A/RTN4A were quantified using Pearson's correlation coefficient. For negative control (Neg) one of the channels from the same ROI was rotated by 90°**.** Bars, 10 µm.

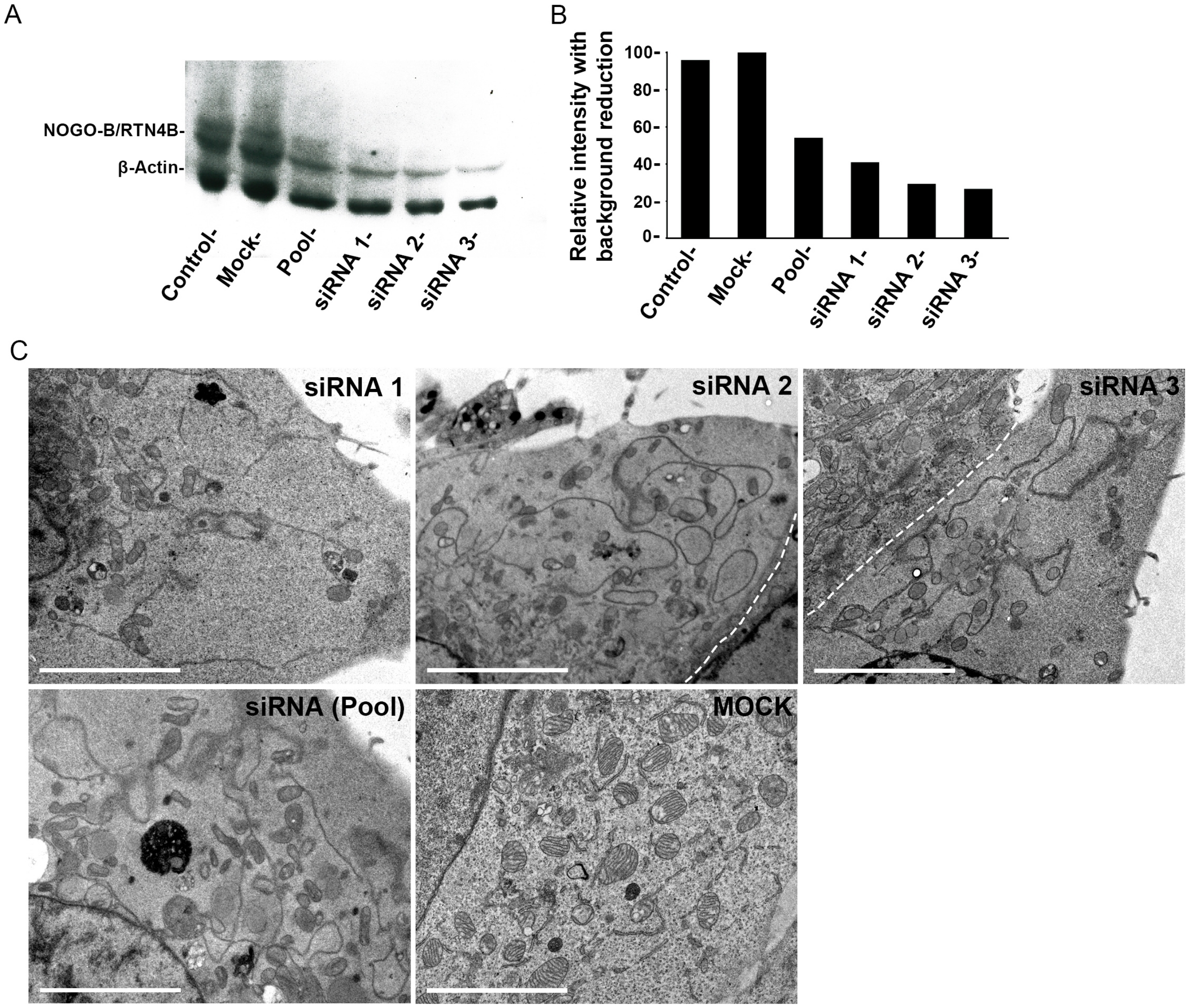

Supplementary Figure S3. Silencing of NOGO-B/RTN4B using siRNAs. (A) Western blot and (B) intensity quantification of NOGO-B/RTN4B in Huh-7 cells after 48 h silencing for three SiRNA individually and for a pool. β-actin was used as a loading control. (C) Thin section TEM images of corresponding cells showing that three silencing constructs yielding similar degree of silencing produce the same morphological effects. Bars, 5 µm.

Supplementary video legends

Supplementary Video S1. 3D model of Huh-7 ER reveal that NOGO-B/RTN4B is localized preferentially on ER tubules and sheet edges. Wild type Huh-7 cells were immunolabelled with anti-NOGO-B/RTB4B antibody. Dual axis tilt series from two consecutive 250-nm thick sections were acquired using SerialEM software running on a Tecnai FEG 20 microscope (FEI). Voxel size is 3.4 x 3.4 x 3.4 nm. Bar 300 nm.

Supplementary Video S2. ER network is organized distinctly in various parts of SCG primary mouse neuron. SB-EM dataset and outlines of the cell (transparent green), nucleus (blue) and ER (yellow) at three different regions (boxes outlined in red). Images were acquired using FEG-SEM Quanta 250 (FEI) equipped with 3View system (Gatan Inc.). Voxel size is 15 x 15 x 30 nm. Bar 10 µm.

Supplementary Video S3. RTN4B-EGFP overexpression induces long ER tubules that are non-motile. Confocal frames of live Huh-7 cells expressing Hsp47-EGFP (left panel) or RTN4B-EGFP (right panel). Images were acquired after 24 hours expression using an inverted TCS SP5II HCS A laser-scanning confocal microscope (Leica). The imaging frame rate was 1 frame/sec and playback frame rate is 10 frame/sec. Bar 10 µm.

Supplementary Video S4. Expression of locked NOGO-B/RTN4B oligomers induces formation of a dense network of short ribosome-free tubules. Huh-7 cells co-expressing NOGO-B/RTN4B - GFP1-10 and NOGO-B/RTN4B - GFP11 constructs were subjected for CLEM. BIFC-signal positive globular profiles in confocal images corresponded with a dense network of short ribosome-free tubules (green) that remained in direct connection with ER sheets (yellow). Dual axis tilt series

from three consecutive 250-nm thick sections were acquired using SerialEM software running on a Tecnai FEG 20 microscope (FEI). Voxel size is 2.3 x 2.3 x 2.3 nm. Bar 1 µm.

Supplementary Video S5. RTN4-depleted Huh-7 cells contain extended fenestrated ER sheets. Electron tomograms and modelled ER (yellow) of a Huh-7 cell RTN4 isoforms were silenced using SiRNA. Dual axis tilt series from three consecutive 250-nm thick sections were acquired using SerialEM software running on a Tecnai FEG 20 microscope (FEI). Voxel size: 2.8 x 2.8 x 2.8 nm. Bar 1 µm.

Supplementary Video S6. RTN4-depletion in Huh-7 cells induces extended stacked ER sheets in cell periphery. SB-EM dataset and modelled ER (yellow) of a Huh-7 cell where RTN4 isoforms were silenced using SiRNA. Images were acquired using FEG-SEM Quanta 250 (FEI) equipped with 3View system (Gatan Inc.). Voxel size is 20 x 20 x 40 nm. Bar 5 µm.

Tables

Table 1. Primer sequences used for qPCR.

**qPCR primer sequences for RTN4 isoforms in hs (Homo Sapiens, Huh-7 cells), mm (Mus musculus, primary mouse cortical neurons (E16))**	
**Primer name**	**Primer sequence (5' to 3')**
hs_*NOGO-A/RTN4A*_fw	GGCTCAGTGGATGAGACCCT
hs_*NOGO-A/RTN4A*_rev	TGTTACCTGGCTGCTCCTTC
hs_ *NOGO-B/RTN4B*_fw	CGGGCTCAGTGGTTGTTGA
hs_ *NOGO-B/RTN4B*_rev	ACTGTCAATGAAAGCAGCAGGA
hs_*NOGO-C/RTN4C*_fw	AAGGACAAGGTTGTTGACCTCC
hs_*NOGO-C/RTN4C*_rev	ACTGTCAATGAAAGCAGCAGGA
hs_*RTN4D*_fw	GATACGCTCCTCTGCAGTTGT
hs_*RTN4D*_rev	ATGAAAGCAGCAGGAATAGGCT
hs_*RTN4E*_fw	GAGCTGGCCGAGTGGAAAA
hs_*RTN4E*_rev	GGGTCTCATCAGAACTCTCTCCT
hs_β-actin_fw	CCAACCGCGAGAAGATGACC
hs_ β-actin_rev	AGAGGCGTACAGGGATAGCA
mm_*Nogo-A/Rtn4A*_fw	GCTCAGTGGATGAGACCCTTTT
mm_*Nogo-A/Rtn4A*_rev	AACAGTGTTACCTGGCTGCT
mm_*Nogo-B/Rtn4B*_fw	CTCGGGCTCAGTGGTTGTT
mm_*Nogo-B/Rtn4B*_rev	ACACTGTCAGAGACAGCAGC
mm_*Nogo-C/Rtn4C*_fw	TACCCTCCTCTGCAGTTGTTG
mm_*Nogo-C/Rtn4C*_rev	GTCAGAGACAGCAGCAGGAATA
mm_*Rtn4D*_fw	TGCATAATTTGTAATTGCTGCTGGA
mm_*Rtn4D*_rev	CAGTACAGGAGGTCAACAACCTT
mm_ *Rtn*4E_fw	GGTGCCTTCATTGTTTGTCGG
mm_*Rtn*4E_rev	GGTCTCATTCCTAGCTGCTGAT
mm_ β-actin_fw	CTAAGGCCAACCGTGAAAAG
mm_ β-actin _rev	ACCAGAGACATACAGGGACA
